# Occurrence and Characterization of Antimicrobial-Resistant and Virulent *Enterococcus* spp. in Dog Feces from Urban Green Spaces in Porto (Portugal)

**DOI:** 10.3390/antibiotics15040379

**Published:** 2026-04-08

**Authors:** Jessica Ribeiro, Rui Lameiras, Vanessa Silva, Gilberto Igrejas, Francisco Cortez Nunes, Ana Isabel Ribeiro, Teresa Letra Mateus, Patrícia Poeta

**Affiliations:** 1 Microbiology and Antibiotic Resistance Team (MicroART), Department of Veterinary Sciences, University of Trás-os-Montes and Alto Douro, 5000-801 Vila Real, Portugal; jessica.ribeiro@ipb.pt (J.R.); ruilameiras04@gmail.com (R.L.); vanessasilva@utad.pt (V.S.); ppoeta@utad.pt (P.P.); 2 Associated Laboratory for Green Chemistry (LAQV-REQUIMTE), Department of Chemistry, University NOVA of Lisbon, 2829-516 Lisbon, Portugal; gigrejas@utad.pt; 3 Centro de Investigação de Montanha (CIMO), La SusTEC, Instituto Politécnico de Bragança, 5300-253 Bragança, Portugal; 4 Functional Genomics and Proteomics Unit, Department of Genetics and Biotechnology, University of Trás-os-Montes and Alto Douro, 5000-801 Vila Real, Portugal; 5 Veterinary and Animal Research Centre (CECAV), University of Trás-os-Montes and Alto Douro, 5000-801 Vila Real, Portugal; 6 Associate Laboratory for Animal and Veterinary Science (AL4AnimalS), University of Trás-os-Montes and Alto Douro, 5000-801 Vila Real, Portugal; 7 Faculty of Medicine and Biomedical Sciences, University of Algarve, 8005-139 Faro, Portugal; franciscojvcnunes@gmail.com; 8 Department of Geography, Faculdade de Letras, Universidade do Porto, Centre of Studies in Geography and Spatial Planning (CEGOT), 4150-564 Porto, Portugal; anaisabelribeiro@letras.up.pt; 9 EPIUnit—Instituto de Saúde Pública, Universidade do Porto, 4050-600 Porto, Portugal; 10 CISAS—Center for Research and Development in Agrifood Systems and Sustainability, Escola Superior Agrária, Instituto Politécnico de Viana do Castelo, 4900-347 Viana do Castelo, Portugal

**Keywords:** antimicrobial resistance, environmental reservoirs, NDVI, One Health, public health risk, socioeconomic deprivation, virulence factors

## Abstract

**Background/Objectives**: *Enterococcus* spp. are important indicators of AMR and potential opportunistic pathogens. Urban green spaces, frequented by dogs and humans, may serve as reservoirs for resistant bacteria. This study assessed the occurrence, AMR profiles, and virulence traits of *Enterococcus* spp. in dog feces from urban green spaces in Porto (Portugal). **Methods**: In December 2023 and May 2024, 240 dog fecal samples were collected from 12 urban green spaces across Porto. *Enterococcus* spp. were isolated using selective culture, identified to species level, and tested for antimicrobial susceptibility following CLSI guidelines. PCR screening was performed for resistance genes (*vanA*, *vanB*, *erm(A/B/C)*, *vatD/E*, *tet(M/O/L/K)*) and virulence genes (*gelE*, *ace*). Environmental and socioeconomic features, including vegetation density (NDVI), presence of water features, and neighborhood deprivation (EDI), were recorded to explore associations with bacterial occurrence and traits. **Results**: Thirty-two isolates were recovered, mainly *E. faecium* (*n* = 9) and *E. faecalis* (*n* = 7). High resistance rates were observed to tetracycline (56.3%) and quinupristin/dalfopristin (37.5%), with lower rates for vancomycin, teicoplanin, and ciprofloxacin (3.1%), and imipenem (6.3%). *Tet(M)* was the most prevalent resistance gene (40.6%), and *gelE* and *ace* were frequently detected, often co-occurring with resistance determinants. Distribution of resistance and virulence genes varied across green spaces, with widely used parks showing more isolates. Vegetation density and water features were not directly associated with bacterial recovery. **Conclusions**: Dog feces in urban green spaces contribute to localized AMR hotspots, acting as potential reservoirs of resistant and potentially pathogenic *Enterococcus* spp. These findings highlight the importance of One Health strategies for urban sanitation and AMR surveillance.

## 1. Introduction

*Enterococcus* spp. are Gram-positive bacteria that naturally inhabit the gastrointestinal tract of humans and animals and are widely used as indicators of fecal contamination in environmental matrices [1,2]. Among the different species, *Enterococcus faecalis* and *Enterococcus faecium* are the most clinically relevant, being responsible for a variety of nosocomial infections [3]. Other species, such as *Enterococcus hirae* and *Enterococcus gallinarum*, are commonly associated with animals and environmental sources, though they are increasingly being identified as causes of human infections [4,5,6,7,8]. The ability of *Enterococcus* to survive under harsh environmental conditions, combined with their intrinsic and acquired resistance mechanisms, makes them efficient reservoirs and disseminators of antimicrobial resistance (AMR) genes in both clinical and non-clinical settings [9].

The rapid emergence of drug-resistant *Enterococcus* strains, particularly those resistant to vancomycin, macrolides, and tetracyclines, represents a significant challenge to public health, as these are employed for human therapies and used in animal production [10]. Resistance to these antimicrobials is often mediated by transferable genetic determinants, such as the *vanA* and *vanB* genes (vancomycin resistance), *erm* genes (macrolide resistance), and *tet* genes (tetracycline resistance) [11,12]. Moreover, virulence factors such as gelatinase (*gelE*) and collagen adhesin (*ace*) contribute to colonization and persistence in the environment and host tissues [13]. The coexistence of these resistance and virulence genes enhances the potential of *Enterococcus* spp. to act as reservoirs and vectors of AMR within the One Health framework, linking human, animal, and environmental health [14,15].

Urban green spaces are increasingly recognized as essential Nature-Based Solutions (NBS) that play a vital role in promoting public health and community well-being [16]. A growing body of evidence links exposure to green spaces to healthier behaviors and improved mental and physical health across the life course—from children to adults—and to their role in buffering the effects of adverse events [17,18,19]. However, for these areas to effectively serve their salutogenic purpose, it is fundamental to ensure they are safe and free from biological hazards. The contamination of urban green spaces by dog feces is not merely an aesthetic issue but a significant public health challenge, as it can transform these recreational areas into reservoirs for the environmental dissemination of antimicrobial-resistant bacteria. In this context, domestic dogs emerge as key agents in the urban ecosystem that contribute to this spread through fecal contamination [20]. Their waste can contaminate soil, water, and vegetation, facilitating the dissemination of antimicrobial-resistant bacteria to humans and other animals through direct or indirect contact [21,22]. Despite growing awareness of this issue, data on antimicrobial resistance in *Enterococcus* spp. of canine origin, particularly from urban environments, remain limited [23].

This study aimed to characterize *Enterococcus* spp. isolated from dog feces collected in urban green spaces of Porto (Portugal), focusing on their antimicrobial susceptibility profiles and the presence of selected resistance (*tet*, *erm*, *van*, *vat*) and virulence (*gelE*, *ace*) genes. In addition to microbiological characterization, the study explored the environmental and urban context of the sampled green spaces by considering ecological and socioeconomic descriptors, allowing a broader interpretation of the factors that may influence the persistence and dissemination of antimicrobial-resistant enterococci in public recreational areas. By integrating microbiological, environmental, and urban dimensions, this work provides insight into the potential role of canine fecal contamination in shaping AMR dynamics in urban green spaces and reinforces the relevance of One Health-based surveillance strategies.

## 2. Results and Discussion

### 2.1. Prevalence of Enterococcus spp.

A total of 240 dog fecal samples were collected from 12 urban green spaces in Porto, with 10 samples obtained per garden in each of the two sampling periods (December 2023 and May 2024). From these samples, 32 *Enterococcus* spp. isolates were recovered, corresponding to an overall isolation rate of 13.3%. While this isolation rate is lower than might be expected based on the natural canine enteric flora, it may reflect natural variability in *Enterococcus* abundance among individual dogs, environmental factors affecting bacterial viability in urban green spaces, and the strict criteria used for selecting only fresh, uncontaminated fecal samples [24]. Care was also taken to minimize the time between collection and laboratory processing. These results indicate that *Enterococcus* spp. are present in dog feces deposited in urban green spaces, supporting their role as indicators of fecal contamination and potential environmental reservoirs of antimicrobial-resistant bacteria in public settings.

**Figure 1:**
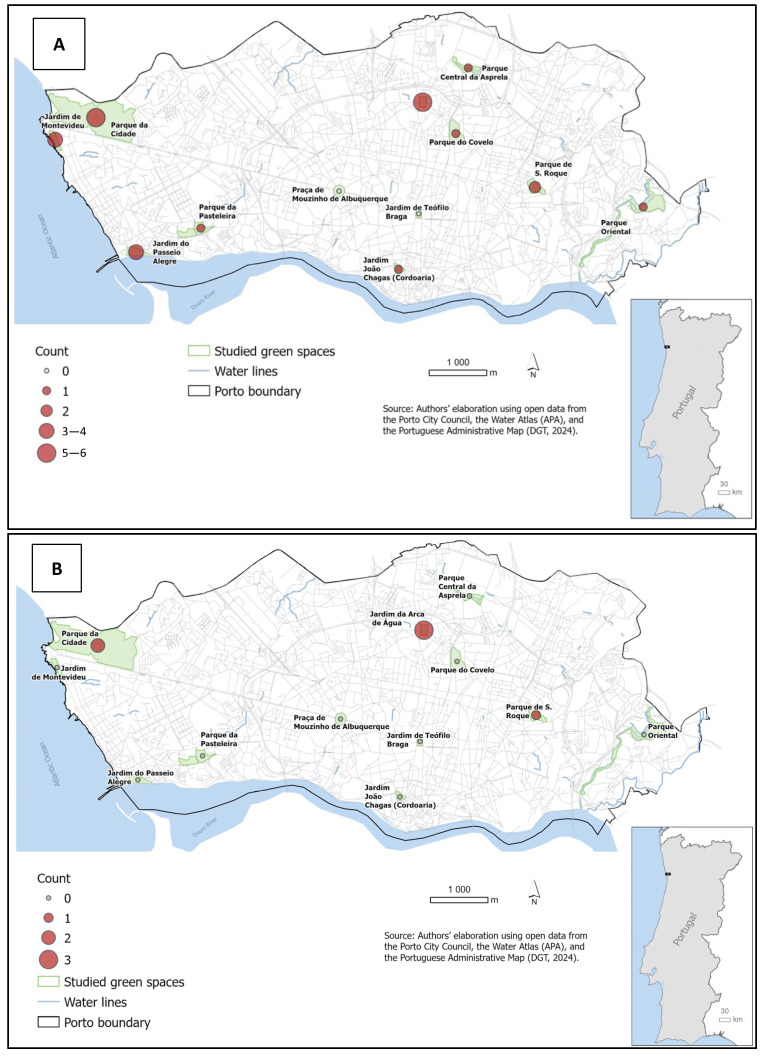
Spatial distribution and prevalence of *Enterococcus* spp. in Porto’s urban green spaces during December 2023 (**A**) and May 2024 (**B**).

The spatial distribution of *Enterococcus* spp. isolates, as illustrated in Figure 1, reveals a widespread occurrence of fecal contamination across the urban landscape of Porto. Considering the sampling effort (10 samples per site and period), recovery rates varied among locations. Higher proportions of positive samples were observed in Jardim da Arca de Água (*n* = 6 in December and *n* = 3 in May), Parque da Cidade (*n* = 5 in December and *n* = 2 in May), and Parque de S. Roque (*n* = 2 in December and *n* = 1 in May). These differences should be interpreted with caution, as recovery rates may be influenced by factors such as sample preservation conditions and environmental exposure. This generalized presence highlights that dog feces serve as a consistent source of environmental dissemination of *Enterococcus* strains throughout the city [25]. Furthermore, the identification of *Enterococcus* spp. in diverse recreational areas highlights the importance of maintaining hygienic practices, such as the proper removal of dog feces, to minimize potential exposure to a range of environmental bacteria [26,27].

Regarding the sampling periods, a relevant variation in the prevalence and diversity of *Enterococcus* species was observed (Table 1). In December, a total of 26 isolates were recovered, with *E. faecium* being the most frequent species (30.8%), followed by *E. faecalis* (11.5%). In contrast, the sampling in May yielded only 6 isolates; however, a shift in species dominance was noted, with *E. faecalis* representing 66.7% of the isolates, while *E. faecium* accounted for only 16.7%. The remaining isolates from both periods (57.7% in December and 16.7% in May) were identified as *Enterococcus* spp., following negative PCR results for *E. hirae* and *E. gallinarum*. The environmental conditions typical of December in Porto, characterized by high humidity levels and moderate temperatures, may enhance the environmental persistence of a wider variety of enterococci in dog feces. These factors protect the bacteria from desiccation, thereby maintaining a higher bacterial load in urban spaces [28]. Conversely, the higher temperatures and increased UV radiation exposure in May might selectively favor more resilient species, such as *E. faecalis*, which represented 66.7% of the isolates in that period, or lead to a faster degradation of fecal matter, resulting in the recovery of fewer but potentially more robust multidrug-resistant strains [29]. This may be explained by the fact that certain antimicrobial resistance mechanisms, such as efflux pumps and stress response systems, can also contribute to enhanced tolerance to environmental stressors, thereby supporting bacterial persistence under adverse conditions [30]. These findings underscore the importance of year-round monitoring and a One Health approach to fully understand how seasonal dynamics influence the role of canine fecal contamination as a persistent source of resistant bacteria in shared public environments.

### 2.2. Antimicrobial Resistance Profiles

**Figure 2:**
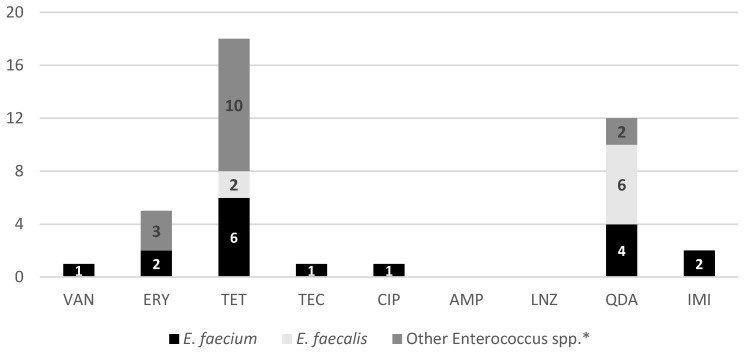
Phenotypic AMR profiles of the 32 *Enterococcus* spp. isolates. The bars represent the number of isolates resistant to each tested antibiotic, categorized by species: *E. faecium* (*n* = 9), *E. faecalis* (*n* = 7), and other *Enterococcus* spp. (*n* = 16). Tested antibiotics include VAN (vancomycin), ERY (erythromycin), TET (tetracycline), TEC (teicoplanin), CIP (ciprofloxacin), AMP (ampicillin), LNZ (linezolid), QDA (quinupristin/dalfopristin), and IMI (imipenem). * Isolates negative for *E. hirae* and *E. gallinarum* by PCR.

The phenotypic analysis of the 32 *Enterococcus* spp. isolates reveal a concerning distribution of AMR within Porto’s urban green spaces (Figure 2). The most striking finding is the high prevalence of resistance to tetracycline, which was observed in 56.3% (*n* = 18) of the isolates, affecting *E. faecium*, *E. faecalis*, and other species alike. Another significant observation is the resistance to quinupristin/dalfopristin, detected in 37.5% (*n* = 12) of the total isolates, with a particularly high occurrence in *E. faecalis* (*n* = 6) and *E. faecium* (*n* = 4). While some resistance in *E. faecalis* can be expected, the presence of these phenotypes in a public space is a matter of public health concern [31]. Furthermore, the study identified resistance to last-resort antibiotics, including a 3.1% (*n* = 1) resistance rate for vancomycin, teicoplanin, and ciprofloxacin, as well as a 6.3% (*n* = 2) resistance rate for imipenem among the isolates. The presence of these resistant phenotypes in dog feces collected from recreational areas suggests that urban green spaces are acting as environmental reservoirs for resistant bacteria. This poses a potential risk of transmission to humans and other animals through contact with contaminated soil or vegetation [20].

The analysis of phenotypic resistance across the two sampling periods suggests potential seasonal differences in the AMR profiles within the urban environment. During the December sampling period, which yielded a higher number of isolates (*n* = 26), a diverse range of resistance profiles was observed, including resistance to tetracycline (53.8%), erythromycin (15.4%), and quinupristin/dalfopristin (26.9%), alongside sporadic resistance to vancomycin, ciprofloxacin, and imipenem (3.8% each). In contrast, although the May collection resulted in fewer isolates (*n* = 6), relatively high proportions of resistance were also observed, with four out of six (66.7%) isolates showing resistance to tetracycline and five (83.3%) exhibiting resistance to quinupristin/dalfopristin. Teicoplanin resistance was detected only once, during the May sampling. These seasonal differences may suggest that while the colder and more humid conditions in December facilitate the environmental persistence of *Enterococcus* strains, the warmer conditions in May might favor the survival of specific, highly resilient multidrug-resistant isolates [32]. However, these observations should be interpreted with caution due to the limited number of isolates, particularly in May, and potential influences of sample condition on isolation success. Therefore, no definitive conclusions regarding seasonal variation can be drawn.

**Figure 3:**
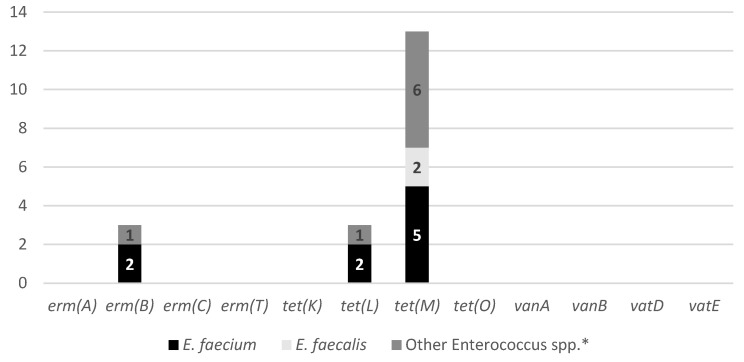
Genotypic AMR profiles of the 32 *Enterococcus* spp. isolates. The bars represent the absolute number of resistance genes detected in each species: *E. faecium* (*n* = 9), *E. faecalis* (*n* = 7), and other *Enterococcus* spp. (*n* = 16). The genetic screening targeted determinants associated with resistance to erythromycin (*erm(A)*, *erm(B)*, *erm(C)*, *erm(T)*), tetracycline (*tet(K)*, *tet(L)*, *tet(M)*, *tet(O)*), vancomycin (*vanA*, *vanB*), and quinupristin-dalfopristin (*vatE*, *vatD*). * Isolates negative for *E. hirae* and *E. gallinarum* by PCR.

The distribution of AMR genes among the *Enterococcus* spp., providing insight into the genetic basis of the resistance phenotypes observed, is presented in Figure 3. The most prevalent resistance determinant was *tet(M)*, identified in 40.6% of the isolates, making it the dominant gene across all *Enterococcus* species analyzed. The predominance of the *tet(M)* gene is particularly concerning as it is frequently associated with conjugative transposons of the Tn916/Tn1545 family, which play a major role in horizontal gene transfer between bacteria from animal, human, and environmental origins [33,34]. The detection of this gene in isolates derived from dog feces deposited in urban green spaces suggests that these environments may function as reservoirs for transferable tetracycline resistance genes. The additional presence of *tet(L)* and *tet(K)* further reflects the diversity of tetracycline resistance mechanisms, including efflux-mediated pathways, circulating in the urban environment. Eflux pumps are transporter proteins that extrude several toxic substances, including antibiotics, from within a cell to its external environment [35]. Macrolide resistance genes were detected at lower frequencies, with *erm(B)* being the only identified (9.3%) *erm* determinant. The coexistence of tetracycline and macrolide resistance genes in some isolates may facilitate co-selection, particularly in environments exposed to multiple antimicrobial pressures [36]. Importantly, no streptogramin resistance genes (*vatD* or *vatE*) were detected, despite the phenotypic resistance to quinupristin–dalfopristin observed in several isolates. Similarly, vancomycin resistance genes (*vanA* and *vanB*) were not identified, even though one isolate exhibited a vancomycin-resistant phenotype. The lack of full correlation between phenotypic and genotypic profiles is a well-documented phenomenon in *Enterococcus* spp. These discrepancies suggest that resistance may be mediated by alternative mechanisms, such as intrinsic resistance traits, chromosomal mutations, or resistance genes not targeted by the PCR assays used in this study [25,37]. Regarding the antibiotics for which no specific resistance genes were screened, such as imipenem and ciprofloxacin, resistance in *Enterococcus* spp. is frequently associated with chromosomal mutations or non-specific mechanisms like efflux pumps, rather than easily detectable mobile genetic elements [38]. Indeed, previous studies have shown that imipenem resistance in *E. faecalis* depends on the presence of a low-affinity PBP4, supporting the rationale for not targeting specific resistance genes for this antibiotic [39]. Additionally, teicoplanin resistance was indirectly assessed through the screening of *vanA* and *vanB* genes, which represent the primary genetic determinants for glycopeptide resistance in this genus [37].

The occurrence of AMR genes demonstrated distinct patterns between the two sampling periods. During the winter sampling (December 2023), the most frequently detected determinant was *tet(M)* (34.6%), followed by *erm(B)* (12.6%) and *tet(L)* (6.3%). In contrast, isolates recovered in the spring sampling (May 2024) exhibited a higher relative contribution of tetracycline resistance genes, with *tet(M)* detected in 66.7% of the isolates and *tet(L)* in 12.6%, while *erm(B)* was identified in only 6.3% of the isolates. Once more, the increase in the proportion of *tet(M)*-positive isolates during the spring period suggests a potential seasonal influence on the environmental burden of tetracycline-resistant *Enterococcus* spp. in Porto’s urban green spaces, possibly related to differences in environmental conditions that favor the persistence or selection of resistant strains [32].

**Figure 4:**
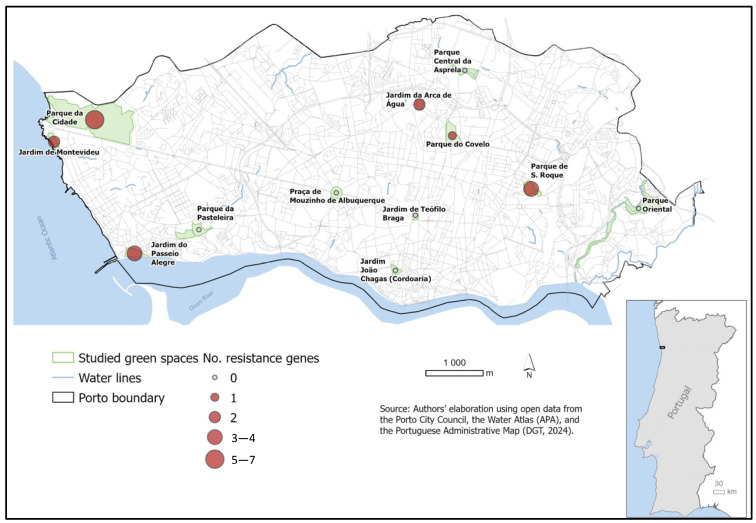
Geographic distribution of AMR genes detected in the 32 *Enterococcus* spp. isolates. The size and color of the markers indicate the total number of resistance genes (including *tet*, *erm*, *van*, and *vat* variants) identified per sampling location.

The geographic distribution of AMR genes (Figure 4) indicates areas with relatively higher genetic diversity, although some regions show very low counts. Locations such as Parque da Cidade, Parque de S. Roque, and Jardim do Passeio Alegre exhibited the highest variety of resistance determinants, harboring variants of *tet* and *erm*. AMR genes in specific green spaces indicate that canine fecal contamination is not only spreading resistant bacteria but also contributing to the accumulation of a complex ‘resistome’ in areas of high human–animal interaction [40].

### 2.3. Virulence Profiles

**Figure 5:**
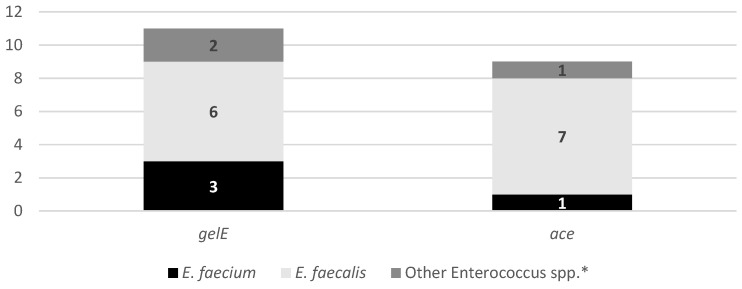
Virulence profiles of the 32 *Enterococcus* spp. isolates. The bars represent the absolute number of virulence factors detected in each species: *E. faecium* (*n* = 9), *E. faecalis* (*n* = 7), and other *Enterococcus* spp. (*n* = 16). The genetic screening targeted determinants associated with *gelE* and *ace*. * Isolates negative for *E. hirae* and *E. gallinarum* by PCR.

**Figure 6:**
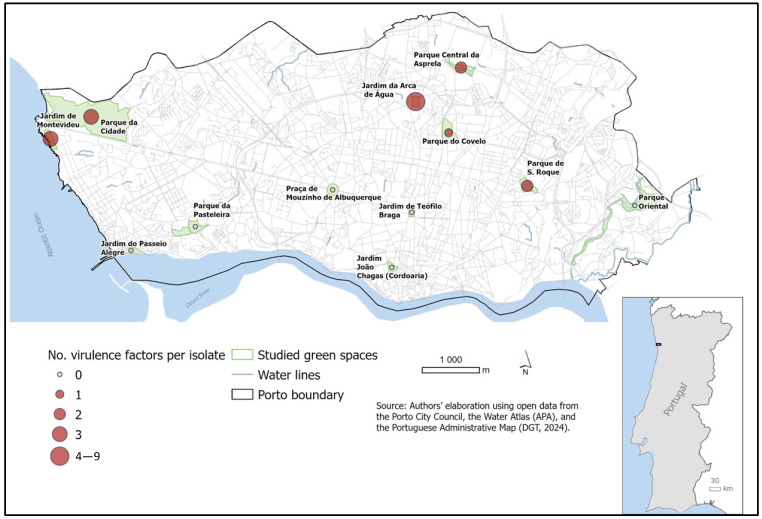
Spatial occurrence and density of virulence-associated genes (*gelE* and *ace*) in *Enterococcus* spp. isolates. Symbols represent the cumulative number of virulence factors per isolate at each sampled urban green space. These findings underscore the potential of canine fecal contamination as a source of clinically relevant strains in shared human–animal environments.

The analysis of virulence-associated genes in *Enterococcus* spp. isolated from dog feces (Figure 5) revealed the presence of clinically relevant determinants in a considerable proportion of isolates. The *gelE* gene was the most frequently detected virulence factor (34.4%), followed by *ace* (28.1%), and both genes were identified across different *Enterococcus* species, including *E. faecium* and *E. faecalis*. The detection of *gelE*, a gene previously associated with tissue degradation, biofilm formation, and environmental persistence, suggests that these isolates may harbor traits that could enhance survival in urban environments and facilitate dissemination [41]. Similarly, the presence of *ace*, a collagen-binding adhesin linked to host colonization and invasive infections, highlights the potential pathogenic relevance of enterococci circulating in urban green spaces [42]. Notably, some isolates harbored more than one virulence determinant, indicating the coexistence of traits that may increase both environmental fitness and pathogenic potential. The occurrence of these virulence genes in *Enterococcus* spp. from dog feces reinforces concerns regarding urban green spaces as reservoirs of opportunistic pathogens. Taken together, the virulence profiles observed in Figure 6, particularly when combined with the AMR data, support the role of canine fecal contamination in the environmental dissemination of enterococci with clinically relevant characteristics, underscoring the importance of surveillance within a One Health framework [43].

A temporal variation in the distribution of virulence-associated genes was observed between the two sampling periods. In December, *gelE* was detected in 7 out of 26 *Enterococcus* spp. isolates (26.9%), while *ace* was identified in four isolates (15.4%), all of which also carried *gelE*. In contrast, despite the lower number of isolates recovered in May (*n* = 6), a higher relative frequency of virulence determinants was observed, with *gelE* detected in four isolates (66.7%) and *ace* in five isolates (83.3%). Notably, all *gelE*-positive isolates recovered in May also harbored *ace*, indicating a predominance of strains carrying combined virulence traits during this period. Again, these findings may indicate that while winter conditions could favor the environmental persistence of a broader diversity of *Enterococcus* spp., spring conditions could selectively allow the persistence of strains with enhanced adhesion and colonization potential [32]. However, these observations should be interpreted with caution due to the limited number of isolates and the potential influence of sample condition on isolation success. Overall, the detection of virulence determinants in both sampling periods highlights dog fecal contamination as a continuous source of potentially pathogenic enterococci in urban green spaces, reinforcing the relevance of year-round monitoring within a One Health framework.

Figure 6 illustrates the spatial distribution and density of virulence-associated genes (*gelE* and *ace*) detected in the 32 *Enterococcus* spp. isolates. Virulence determinants were not restricted to a single location but were detected in multiple green spaces, indicating a widespread dissemination of potentially pathogenic enterococci throughout the urban environment. The co-occurrence of *gelE* and *ace* in several locations further highlights the presence of strains with combined adhesion and tissue-degrading capabilities, which may enhance environmental persistence and increase the likelihood of host colonization following exposure. The spatial overlap between virulence gene hotspots and areas previously identified as harboring AMR determinants reinforces the concern that certain urban green spaces may function as reservoirs of enterococci with both virulence and resistance traits. This convergence increases the potential public health risk, as it facilitates the circulation of strains with enhanced fitness and pathogenic potential at the human–animal–environment interface.

### 2.4. Ecological and Urban Drivers of Antimicrobial-Resistant Enterococcus spp.

The analysis of vegetation density across the sampled urban green spaces, assessed using the mean Normalized Difference Vegetation Index (NDVI), revealed some heterogeneity among the public green spaces of Porto. Mean NDVI values ranged from 0.229 in Parque de S. Roque to 0.495 in Jardim João Chagas (Cordoaria), reflecting differences in vegetation coverage, structure, and vigor across the studied locations. However, when NDVI values were examined in relation to the spatial distribution of *Enterococcus* spp. isolates and their associated AMR and virulence determinants, no direct positive association between higher vegetation density and increased bacterial recovery was observed. Among the green spaces with higher NDVI values (mean NDVI > 0.40), such as Jardim de Montevideu, Praça de Mouzinho de Albuquerque, Jardim de Teófilo Braga, and Jardim João Chagas (Cordoaria), only Jardim de Montevideu yielded *Enterococcus* spp. isolates and corresponding resistance and virulence genes. In contrast, the highest numbers of isolates were recovered from Jardim da Arca de Água (*n* = 6 in December; *n* = 3 in May), Parque da Cidade (*n* = 5 in December; *n* = 2 in May), and Parque de S. Roque (*n* = 2 in December; *n* = 1 in May), which are not among the sites with the highest NDVI values. From an ecological perspective, highly vegetated green spaces are typically associated with shaded microhabitats, increased soil moisture, and reduced exposure to ultraviolet radiation, which are environmental conditions known to favor bacterial survival in soil and on vegetation surfaces [44,45]. Such conditions may enhance the persistence of fecal material deposited by companion animals and potentially prolong the environmental survival of antimicrobial-resistant *Enterococcus* spp. Nevertheless, the present findings indicate that vegetation density does not constitute a direct risk factor for bacterial occurrence per se but may instead act as an ecological facilitator by modulating microenvironmental conditions (such as moisture retention, organic matter availability, and host or vector presence) whose influence is strongly context dependent. Factors such as the intensity of human and animal activity, repeated fecal inputs, soil disturbance, and local management practices are likely to modulate the extent to which favorable microclimatic conditions translate into detectable bacterial persistence [46].

In addition to vegetation density, other characteristics of the sampled green spaces, including the park size and presence of water features, were considered to contextualize the environmental distribution of *Enterococcus* spp. and their associated resistance and virulence determinants. Large and highly frequented green spaces, such as Parque da Cidade, combine extensive surface area, diverse vegetation structures, and multiple water features, creating heterogeneous microhabitats that may support the persistence of antimicrobial-resistant enterococci. Conversely, smaller and more compact green spaces, including Jardim João Chagas (Cordoaria) and Praça de Mouzinho de Albuquerque, are subject to a higher intensity of use per unit area, which may increase fecal deposition pressure and contribute to the detection of *Enterococcus* spp. despite their limited spatial extent. Among the sites where *Enterococcus* spp. were recovered, Parque da Cidade and Parque de S. Roque are characterized by the presence of lakes or fountains within their boundaries. However, bacterial isolates were also recovered from Parque do Covelo during the December sampling period, despite the absence of both natural watercourses and artificial water features at this site. Conversely, Jardim João Chagas (Cordoaria), which includes artificial water elements, did not yield *Enterococcus* spp. isolates during the May sampling campaign. These contrasting observations suggest that the presence or absence of water bodies alone is insufficient to explain the spatial and temporal distribution of *Enterococcus* spp. in urban green spaces.

To further contextualize the environmental distribution of *Enterococcus* spp., the socioeconomic characteristics of the areas surrounding each green space were assessed using the European Deprivation Index (EDI). Based on the distribution of EDI values across the sampled locations, sites were categorized into low, medium, and high deprivation using empirically defined thresholds (EDI < −3, −3 ≤ EDI ≤ 2, EDI > 2, respectively). A socioeconomic heterogeneity was observed among the sampled green spaces. Jardim de Montevideu, Parque da Cidade, Praça de Mouzinho de Albuquerque, and Jardim do Passeio Alegre were classified as low-deprivation areas; Parque do Covelo, Parque de S. Roque, Jardim da Arca de Água, Jardim de Teódilo Braga, Parque Central da Asprela, and Parque da Pasteleira as medium-deprivation áreas; and Jardim João Chagas (Cordoaria) and Parque Oriental as high-deprivation areas. Most *Enterococcus* spp. isolates were recovered from green spaces located in low- and medium-deprivation neighborhoods, whereas only a single isolate was obtained from each of the two high-deprivation sites, Parque João Chagas (Cordoaria) and Parque Oriental, during the December sampling. Importantly, these isolates did not carry AMR or virulence-associated genes, indicating that socioeconomic deprivation alone does not necessarily favor the persistence of resistant or virulent enterococci in urban green spaces. A possible explanation is that lower access to veterinary care in these high-deprivation areas may result in less frequent antibiotic use in pets, reducing selective pressure for the emergence and dissemination of resistant strains [47].

Although the exploratory nature of this study and the limited number of sampling sites preclude causal inference, integrating socioeconomic indicators with ecological and microbiological data provides a more comprehensive understanding of AMR dissemination in urban environments [48]. The combined assessment of vegetation density, water features, and spatial dimensions suggests that certain urban green spaces create ecological conditions favorable to the environmental persistence of *Enterococcus* spp. carrying resistance and virulence determinants. Rather than acting as isolated risk factors, these environmental characteristics likely interact to form localized “hotspots” where resistant bacteria may persist and accumulate [49]. Overall, these findings highlight the importance of incorporating biophysical and ecological descriptors into AMR surveillance, as the integration of remotely sensed vegetation indices, spatial metrics, and water feature information allows for a nuanced understanding of how urban green spaces, designed to promote health and well-being, may inadvertently serve as reservoirs for clinically relevant bacteria when pet waste management is insufficient. These results also underscore the value of including social context in One Health surveillance frameworks, recognizing that AMR in public spaces emerges from the dynamic interplay between environmental structure, animal behavior, and broader urban inequalities.

## 3. Materials and Methods

### 3.1. Sample Collection

**Figure 7:**
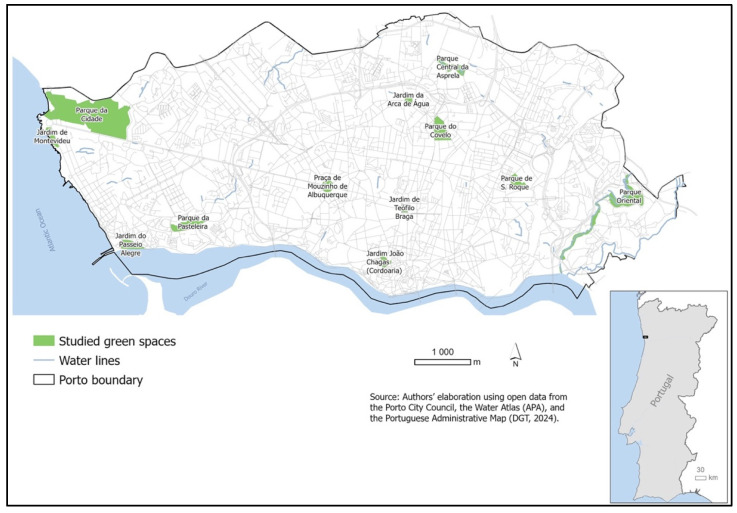
Geographic location of the 12 urban green spaces sampled in Porto (Portugal). Sampling sites were selected to represent different urban areas for the collection of dog fecal samples (*n* = 240) during the two study periods (December 2023 and May 2024).

Fecal samples were collected in December 2023 and May 2024 from 12 urban green spaces located across the city of Porto (Portugal): Jardim de Montevideu, Parque da Cidade, Jardim do Passeio Alegre, Parque da Pasteleira, Praça de Mouzinho de Albuquerque, Jardim de Teófilo Braga, Jardim João Chagas (Cordoaria), Parque de S. Roque, Parque Oriental, Parque do Covelo, Parque Central da Asprela, and Jardim da Arca de Água (Figure 7). Sampling was conducted in two different periods to capture potential seasonal variation. In each garden, ten fresh fecal samples were collected per sampling period, resulting in a total of 240 samples. Only fresh samples, showing no signs of degradation or environmental contamination, were collected to avoid overgrowth of environmental bacteria or cross-contamination. Samples were collected directly from the ground using sterile spatulas and placed into sterile containers. Each sample was labeled according to the collection site and period and transported to the laboratory under refrigerated conditions (4 °C) within 24 h. Upon arrival, samples were processed immediately or stored at 4 °C for a maximum of 48 h before microbiological analysis.

### 3.2. Isolation and Identification of Enterococcus spp.

Approximately 2–3 g of each fecal sample were inoculated into Brain Heart Infusion (BHI) broth (Oxoid Ltd., Basingstoke, UK) and incubated at 37 °C for 24 h to allow bacterial enrichment. After incubation, aliquots from each culture were streaked onto Slanetz–Bartley agar (Oxoid Ltd., Basingstoke, UK) plates and incubated at 37 °C for 48 h. Colonies displaying the typical pink to red coloration characteristic of *Enterococcus* spp. were selected and subsequently subcultured on Kanamycin Aesculin Azide (KAA) agar (Oxoid Ltd., Basingstoke, UK). Plates were incubated at 37 °C for 24 h, and colonies that produced blackening of the medium, indicative of aesculin hydrolysis, were presumptively identified as *Enterococcus* spp. Representative isolates were preserved in skim milk medium at −80 °C for further analyses, including antimicrobial susceptibility testing and PCR detection of selected resistance and virulence genes.

### 3.3. Antimicrobial Susceptibility Testing

Antimicrobial susceptibility was determined by the disk diffusion method on Mueller–Hinton agar (Oxoid Ltd., Basingstoke, UK). The following antibiotics disks (Oxoid Ltd., Basingstoke, UK) were tested (µg per disk): vancomycin (30), teicoplanin (30), erythromycin (15), tetracycline (30), ciprofloxacin (5), ampicillin (10), linezolid (30), quinupristin-dalfopristin (5), and imipenem (10), according to Clinical and Laboratory Standards Institute (CLSI) guidelines [50], except for teicoplanin, quinupristin-dalfopristin, and imipenem, which were included based on the EUCAST guidelines [51]. Plates were incubated at 37 °C for 24 h, and inhibition zone diameters were measured and interpreted according to CLSI and EUCAST breakpoints for *Enterococcus* spp. (Table 2). Isolates exhibiting resistance to at least one antibiotic in three or more antimicrobial classes were classified as multidrug-resistant.

### 3.4. Detection of Antimicrobial Resistance and Virulence Genes

Genomic DNA was isolated from overnight BHI broth cultures using the Insta Gene™ Matrix (Bio-Rad Laboratories, Hercules, CA, USA). DNA concentration and purity were determined with a NanoDrop ND-100 spectrophotometer (Thermo Fisher Scientific, Waltham, MA, USA). Gene detection was then conducted via polymerase chain reaction (PCR). PCR was performed on a ProFlex™ PCR System (Applied Biosystems, Waltham, MA, USA) in a total volume of 50 µL, containing 30.2 µL ultrapure water (Milli-Q^®^, Merck Millipore, Burlington, MA, USA), 5 µL complete buffer (Bioron GmbH, Römerberg, Germany), 1.5 µL 100 mM MgCl_2_ (100 mM, Bioron GmbH, Römerberg, Germany), (10 mM, Bioron GmbH, Römerberg, Germany), 1 µL of each primer (50 µM, Eurofins Genomics, Ebersberg, Germany), 0.3 µL DFS-Taq DNA polymerase (5 U/µL, Bioron GmbH, Römerberg, Germany), and 10 µL of template DNA (10 ng). Positive controls consisted of strains from the MicroART collection, while Milli-Q water was used as the negative control. The analysis focused on four *Enterococcus* species: *E. faecium* (ddl*_E. faecium_*), *E. faecalis* (ddl*_E. faecalis_*), *E. hirae (murG)*, and *E. gallinarum (vanC1)*. Twelve primers were employed to identify genes conferring resistance to five antibiotic classes, including glycopeptides (*vanA*, *vanB*), macrolides (*erm(A)*, *erm(B)*, *erm(C)*) streptogramins (*vatD*, *vatE*), and tetracyclines (*tet(M)*, *tet(O)*, *tet(L)*, *tet(K)*). In addition, isolates were screened for the virulence-associated genes *gelE* (encoding gelatinase) and *ace* (encoding a collagen-binding adhesin). Details on primer sequences, PCR conditions, and expected amplicon sizes are presented in Table 3.

### 3.5. Data Analysis

The frequencies of resistance phenotypes and genotypes were calculated and expressed as percentages. The distribution of resistant isolates and detected genes was analyzed according to *Enterococcus* species and garden location.

Spatial data visualization was performed using ArcGIS Pro 3.5.0. Graduated symbol maps (Esri Inc., Redlands, CA, USA) (natural breaks classification) were produced to depict the prevalence of *Enterococcus* spp. and the distribution of specific AMR and virulence genes across the sampled public urban green spaces in Porto.

In addition, we characterized the features of each green space, namely the presence of water features, vegetation levels, and the socioeconomic deprivation of the surrounding areas. Spatial datasets and analytical procedures were employed.

To assess the presence of water features, we used official cartographic data on surface water bodies provided by the Porto City Council [59]. Spatial overlay operations were conducted to identify whether water bodies intersected each urban green space. The presence of artificial water features (e.g., ponds, fountains, lakes) was further assessed through visual inspection of high-resolution aerial imagery in Google Earth. Vegetation was characterized using the NDVI, computed from Sentinel-2 imagery with less than 5% cloud cover, following the methodology adopted in previous studies [60,61] for the years 2023 and 2024. NDVI was calculated as:

NDVI = (NIR + Red)/(NIR − Red),

where NIR corresponds to the near-infrared band and Red to the red band of the electromagnetic spectrum. NDVI values range from −1 to +1, with higher values indicating greater vegetation density and vigor, and values close to zero or negative indicating sparse or absent vegetation.

Finally, socioeconomic deprivation in the surroundings of each green space was assessed using the EDI, computed for Portuguese small areas, following the methodology fully described elsewhere [62]. A 300-m street-network buffer, corresponding to the distance recommended by the World Health Organization (WHO) as an indicator of adequate accessibility to urban green spaces [63], was generated around each green space to represent its local catchment area. The weighted average EDI was then calculated based on the population of each census block group intersecting the buffer, allowing for a population-adjusted estimate of neighborhood deprivation.

## 4. Conclusions

This study demonstrates that dog feces deposited in urban green spaces represent a relevant environmental reservoir of antimicrobial-resistant and potentially virulent *Enterococcus* spp. in Porto. Although the overall isolation rate was moderate, the recovery of clinically significant species, particularly *E. faecium* and *E. faecalis*, together with the detection of resistance to important antimicrobials, highlights the public health relevance of canine fecal contamination in shared recreational environments.

The high prevalence of tetracycline resistance, largely associated with the widespread detection of the mobile gene *tet(M)*, underscores the role of urban environments as reservoirs and potential dissemination centers for transferable AMR determinants. The presence of virulence-associated genes (*gelE* and *ace*), often co-occurring with resistance traits, further emphasizes the pathogenic potential and environmental fitness of enterococci circulating in public green spaces.

By integrating microbiological findings with ecological and socioeconomic descriptors, this work provides novel insight into the complex drivers shaping the environmental persistence of antimicrobial-resistant bacteria in urban settings. Rather than acting as isolated risk factors, vegetation structure, water features, intensity of human–animal interaction, and neighborhood socioeconomic context appear to co-occur in specific urban green spaces, potentially contributing to localized conditions that may favor the persistence and accumulation of resistant and virulent enterococci. Notably, green spaces located in low- and medium-deprivation areas exhibited a higher diversity of resistance and virulence determinants, suggesting that patterns of park use, pet ownership, and maintenance practices may play a more decisive role than deprivation alone.

Overall, these findings reinforce the concept that urban green spaces, while essential for promoting health and well-being, may inadvertently function as reservoirs for antimicrobial-resistant bacteria when pet waste management is inadequate. This study highlights the importance of adopting integrated One Health surveillance strategies that combine microbiological monitoring with environmental and social indicators. Strengthening responsible pet ownership, improving sanitation practices, and implementing spatially informed monitoring approaches are essential steps to mitigate the environmental dissemination of antimicrobial-resistant and virulent bacteria in urban public spaces.

## Figures and Tables

**Table 1:** Distribution of *Enterococcus* species by sampling period (December 2023 and May 2024).

Species	December (*n* = 26)	May (*n* = 6)	Total (*n* = 32)
*E. faecium*	8 (30.8%)	1 (16.7%)	9 (28.1%)
*E. faecalis*	3 (11.5%)	4 (66.7%)	7 (21.9%)
Other *Enterococcus* spp. ^1^	15 (57.7%)	1 (16.7%)	16 (50.0%)
Total	26 (100%)	6 (100%)	32 (100%)

^1^ Isolates negative for *E. hirae* and *E. gallinarum* by PCR.

**Table 2:** Cut-off values for disk diffusion testing of *Enterococcus* spp. isolates.

Antibiotic	Disk (µg)	Susceptible (S)	Resistant (R)	Reference
Ampicillin	10	≥17 mm	≤16 mm	[50]
Vancomycin	30	≥17 mm	≤14 mm	[50]
Teicoplanin	30	≥16 mm	≤16 mm	[51]
Erythromycin	15	≥23 mm	≤13 mm	[50]
Tetracycline	30	≥19 mm	≤14 mm	[50]
Ciprofloxacin	5	≥21 mm	≤15 mm	[50]
Linezolid	30	≥23 mm	≤20 mm	[50]
Quinupristin-dalfopristin	5	≥22 mm (*E. faecium*)	<20 mm (*E. faecium*)	[51]
Imipenem	10	≥50 mm	<21 mm	[51]

**Table 3:** List of primers employed for PCR-based detection of *Enterococcus* species, antibiotic resistance determinants, and virulence-associated genes. The table provides the target genes, corresponding primer sequences, PCR cycling parameters, and expected product sizes.

Target Gene	Primer Sequence (5′–3′)	Conditions	Size (bp)	Ref.
*ddl_E. faecalis_*	F: ATC AAG TAC AGT TAG TCT R: ACG ATT CAA AGC TAA CTG	94 °C 2 min 94 °C 1 min, 46.9 °C 1 min, 72 °C 1 min (30×) 72 °C 10 min	941	[52] ^1^
*ddl_E. faecium_*	F: TAG AGA CAT TGA ATA TGC R: CTA ACA TCG TGT AAG CT	94 °C 2 min 94 °C 1 min, 50 °C 1 min, 72 °C 1 min (30×) 72 °C 10 min	550	[52] ^1^
*murG* *(E. hirae)*	F: GGC ATA TTT ATC CAG CAC TAG R: CTC TGG ATC AAG TCC ATA AGT GG	94 °C 2 min 94 °C 1 min/55 °C 2 min/72 °C 3 min (35×) 72 °C 10 min	521	[53]
*vanC1* *(E. gallinarum)*	F: GGT ATC AAG GAA ACC TC R: CTT CCG CCA TCA TAG CT	94 °C 2 min 94 °C 1 min/54 °C 1 min/72 °C 1 min (30×) 72 °C 10 min	822	[52]
*vanA*	F: GGG AAA ACG ACA ATT GC R: GTA CAA TGC GGC CGT TA	94 °C 2 min 94 °C 1 min/54 °C 1 min/72 °C 1 min (30×) 72 °C 10 min	732	[52]
*vanB*	F: ATG GGA AGC CGA TAG TC R: GAT TTC GTT CCT CGA CC		635	
*erm(A)*	F: TCT AAA AAG CAT GTA AAA GAA R: CTT CGA TAG TTT ATT AAT ATT AGT	93 °C 3 min 93 °C 1 min/52 °C 1 min/72 °C 1 min (35×) 72 °C 5 min	645	[54]
*erm(B)*	F: GAA AAG ATA CTC AAC CAA ATA R: AGT AAC GGT ACT TAA ATT GTT TAC		639	
*erm(C)*	F: TCA AAA CAT AAT ATA GAT AAA R: GCT AAT ATT GTT TAA ATC GTC AAT		642	
*vatD*	F: CCG AAT CCT ATG AAA ATG TAT CC R: GCA GCTACTATTGCACCATCCC	94 °C 2 min 94 °C 1 min/55 °C 1 min/72 °C 3 min (40×) 72 °C 5 min	413	[55]
*vatE*	F: ACG TTA CCC ATC ACT ATG R: GCT CCG ATA ATG GCA CCG AC		282	
*tet(K)*	F: TTA GGT GAA GGG TTA GGT CC R: GCA AAC TCA TTC CAG AAG CA	94 °C 1 min 94 °C 1 min/55 °C 2 min/72 °C 2 min (30×) 72 °C 10 min	697	[56]
*tet(L)*	F: CAT TTG GTC TTA TTG GAT CG R: ATT ACA CTT CCG ATT TCG G	94 °C 1 min 94 °C 1 min/50 °C 1 min/72 °C 1 min (30×) 72 °C 10 min	456	
*tet(M)*	F: GTT AAA TAG TGT TCT TGG AG R: CTA AGA TAT GGC TCT AAC AA	94 °C 1 min 94 °C 1 min/55 °C 2 min/72 °C 2 min (30×) 72 °C 10 min	576	
*tet(O)*	F: ACG GAR AGT TTA TTG TAT ACC R: TGG CGT ATC TAT AAT GTT GAC	94 °C 5 min 94 °C 30 s/60 °C 30 s/72 °C 30 s (25×) 72 °C 7 min	171	[57]
*gelE*	F: AGT TCA TGT CTA TTT TCT TCA C R: CTT CAT TAT TTA CAC GTT TG	94 °C 3 min 94 °C 1 min/56 °C 1 min/72 °C 1 min (30×) 72 °C 5 min	402	[58]
*ace*	F: AAA GTA GAA TTA GAT CCA CAC R: TCT ATC ACA TTC GGT TGC G		320	

^1^ annealing temperature adapted from the cited source.

## Data Availability

The original contributions presented in this study are included in the article. Further inquiries can be directed to the corresponding author.

## Abbreviations

The following abbreviations are used in this manuscript:

AMP	Ampicillin
AMR	Antimicrobial Resistance
BHI	Brain Heart Infusion
CIP	Ciprofloxacin
CLSI	Clinical and Laboratory Standards Institute
DNA	Deoxyribonucleic Acid
EDI	European Deprivation Index
ERY	Erythromycin
IMI	Imipenem
KAA	Kanamycin Aesculin Azide
LNZ	Linezolid
NBS	Nature-Based Solutions
NDVI	Normalized Difference Vegetation Index
PCR	Polymerase Chain Reaction
QDA	Quinupristin-dalfopristin
SB	Stanetz-Bartley
TEC	Teicoplanin
TET	Tetracycline
UV	Ultra-Violet
VAN	Vancomycin
WHO	World Health Organization

## References

[1] Novais C., Freitas A.R., Silveira E., Antunes P., Silva R., Coque T.M., Peixe L. (2013). Spread of Multidrug-Resistant *Enterococcus* to Animals and Humans: An Underestimated Role for the Pig Farm Environment. J. Antimicrob. Chemother..

[2] Krawczyk B., Wityk P., Gałęcka M., Michalik M. (2021). The Many Faces of *Enterococcus* spp.—Commensal, Probiotic and Opportunistic Pathogen. Microorganisms.

[3] Ayobami O., Willrich N., Reuss A., Eckmanns T., Markwart R. (2020). The Ongoing Challenge of Vancomycin-Resistant *Enterococcus faecium* and *Enterococcus faecalis* in Europe: An Epidemiological Analysis of Bloodstream Infections. Emerg. Microbes Infect..

[4] Avberšek J., Mićunović J., Šemrov N., Ocepek M. (2021). Surveillance of the Source of Poultry Infections with *Enterococcus hirae* and *Enterococcus cecorum* in Slovenia and *E. hirae* Antibiotic Resistance Patterns. New Microbiol..

[5] Coccitto S.N., Cinthi M., Fioriti S., Morroni G., Simoni S., Vignaroli C., Garofalo C., Mingoia M., Brenciani A., Giovanetti E. (2021). Linezolid-Resistant *Enterococcus gallinarum* Isolate of Swine Origin Carrying *cfr*, *optrA* and *poxtA* Genes. J. Antimicrob. Chemother..

[6] Soares R., Miranda C., Cunha S., Ferreira L., Martins Â., Igrejas G., Poeta P. (2023). Antibiotic Resistance of *Enterococcus* Species in Ornamental Animal Feed. Animals.

[7] Zhao B., Ye M.S., Zheng R. (2018). *Enterococcus gallinarum* Meningitis: A Case Report and Literature Review. BMC Infect. Dis..

[8] Piccinini D., Bernasconi E., Di Benedetto C., Martinetti Lucchini G., Bongiovanni M. (2023). *Enterococcus hirae* Infections in the Clinical Practice. Infect. Dis..

[9] Geraldes C., Tavares L., Gil S., Oliveira M. (2022). *Enterococcus* Virulence and Resistant Traits Associated with Its Permanence in the Hospital Environment. Antibiotics.

[10] Sparo M., Delpech G., Allende N.G. (2018). Impact on Public Health of the Spread of High-Level Resistance to Gentamicin and Vancomycin in Enterococci. Front. Microbiol..

[11] Maleki D., Manouchehrifar M., Kheljan M.N., Mossavi S.H., Jannati E., Doghaheh H.P., Teimourpour R., Khademi F., Arzanlou M. (2021). Vancomycin-Resistant *Enterococcus* Species: Antimicrobial Resistance and Virulence Genes Profile. Gene Rep..

[12] Fatoba D.O., Amoako D.G., Akebe A.L.K., Ismail A., Essack S.Y. (2022). Genomic Analysis of Antibiotic-Resistant *Enterococcus* spp. Reveals Novel Enterococci Strains and the Spread of Plasmid-Borne *tet(M)*, *tet(L)* and *erm(B)* Genes from Chicken Litter to Agricultural Soil in South Africa. J. Environ. Manag..

[13] Too E., Masila E., Téllez-Isaías G., Graham D., El-Ashram S. (2024). The Interconnection between Virulence Factors, Biofilm Formation, and Horizontal Gene Transfer in *Enterococcus*: A Review. Enterococcus—Unveiling the Emergence of a Potent Pathogen.

[14] Marques J.M., Coelho M., Santana A.R., Pinto D., Semedo-Lemsaddek T. (2023). Dissemination of Enterococcal Genetic Lineages: A One Health Perspective. Antibiotics.

[15] Zaheer R., Cook S.R., Barbieri R., Goji N., Cameron A., Petkau A., Polo R.O., Tymensen L., Stamm C., Song J. (2020). Surveillance of *Enterococcus* spp. Reveals Distinct Species and Antimicrobial Resistance Diversity across a One-Health Continuum. Sci. Rep..

[16] Pinto L.V., Inácio M., Pereira P. (2023). Green and Blue Infrastructure (GBI) and Urban Nature-Based Solutions (NbS) Contribution to Human and Ecological Well-Being and Health. Oxf. Open Infrastruct. Health.

[17] Ribeiro A.I., Triguero-Mas M., Jardim Santos C., Gómez-Nieto A., Cole H., Anguelovski I., Silva F.M., Baró F. (2021). Exposure to Nature and Mental Health Outcomes during COVID-19 Lockdown. A Comparison between Portugal and Spain. Environ. Int..

[18] Ribeiro A.I., Behlen M., Henriques A., Severo M., Jardim Santos C., Barros H. (2024). Exposure to Green and Blue Spaces and Depression among Older Adults from the EPIPorto Cohort: Examining Environmental, Social, and Behavioral Mediators and Varied Space Types. Cities Health.

[19] Marcilla-Toribio I., Bizzozero-Peroni B., Notario-Pacheco B., Ribeiro A.I., Santos M.P., Fernandez-Perez M., Cekrezi S., la Cruz L.L.d., Martinez-Andres M. (2025). The Role of Surrounding Residential Greenness in Healthy Behaviours: A Systematic Review and Meta-Analysis. Discov. Public Health.

[20] Vassallo A., Kett S., Purchase D., Marvasi M. (2022). The Bacterial Urban Resistome: Recent Advances. Antibiotics.

[21] Gwenzi W., Chaukura N., Muisa-Zikali N., Teta C., Musvuugwa T., Rzymski P., Abia A.L.K. (2021). Insects, Rodents, and Pets as Reservoirs, Vectors, and Sentinels of Antimicrobial Resistance. Antibiotics.

[22] Cinquepalmi V., Monno R., Fumarola L., Ventrella G., Calia C., Greco M.F., De Vito D., Soleo L. (2013). Environmental Contamination by Dog’s Faeces: A Public Health Problem?. Int. J. Environ. Res. Public Health.

[23] Miranda C., Silva V., Igrejas G., Poeta P. (2021). Impact of European Pet Antibiotic Use on Enterococci and Staphylococci Antimicrobial Resistance and Human Health. Future Microbiol..

[24] Lebreton F., Willems R.J.L., Gilmore M.S., Gilmore M.S., Clewell D.B., Ike Y., Shankar N. (2014). *Enterococcus* Diversity, Origins in Nature, and Gut Colonization. Enterococci: From Commensals to Leading Causes of Drug Resistant Infection.

[25] Stępień-Pyśniak D., Bertelloni F., Dec M., Cagnoli G., Pietras-Ożga D., Urban-Chmiel R., Ebani V.V. (2021). Characterization and Comparison of *Enterococcus* spp. Isolates from Feces of Healthy Dogs and Urine of Dogs with UTIs. Animals.

[26] Selway C.A., Mills J.G., Weinstein P., Skelly C., Yadav S., Lowe A., Breed M.F., Weyrich L.S. (2020). Transfer of Environmental Microbes to the Skin and Respiratory Tract of Humans after Urban Green Space Exposure. Environ. Int..

[27] Ibekwe A.M., Obayiuwana A.C., Murinda S.E. (2024). *Enterococcus* Species and Their Antimicrobial Resistance in an Urban Watershed Affected by Different Anthropogenic Sources. Water..

[28] Byappanahalli M.N., Nevers M.B., Korajkic A., Staley Z.R., Harwood V.J. (2012). Enterococci in the Environment. Microbiol. Mol. Biol. Rev..

[29] Raza S., Matuła K., Karoń S., Paczesny J. (2021). Resistance and Adaptation of Bacteria to Non-antibiotic Antibacterial Agents: Physical Stressors, Nanoparticles, and Bacteriophages. Antibiotics.

[30] Dawan J., Ahn J. (2022). Bacterial Stress Responses as Potential Targets in Overcoming Antibiotic Resistance. Microorganisms.

[31] Namaki Kheljan M., Teymorpour R., Peeri Doghaheh H., Arzanlou M. (2022). Antimicrobial Biocides Susceptibility and Tolerance-Associated Genes in *Enterococcus faecalis* and *Enterococcus faecium* Isolates Collected from Human and Environmental Sources. Curr. Microbiol..

[32] MacFadden D.R., McGough S.F., Fisman D., Santillana M., Brownstein J.S. (2018). Antibiotic Resistance Increases with Local Temperature. Nat. Clim. Change.

[33] Agersø Y., Pedersen A.G., Aarestrup F.M. (2006). Identification of Tn5397-like and Tn916-like Transposons and Diversity of the Tetracycline Resistance Gene *tet(M)* in Enterococci from Humans, Pigs and Poultry. J. Antimicrob. Chemother..

[34] Rizzotti L., Gioia F., Dellaglio F., Torriani S. (2009). Molecular Diversity and Transferability of the Tetracycline Resistance Gene *tet(M)*, Carried on Tn916-1545 Family Transposons, in Enterococci from a Total Food Chain. Antonie Leeuwenhoek Int. J. Gen. Mol. Microbiol..

[35] Molale L.G., Bezuidenhout C.C. (2016). Antibiotic Resistance, Efflux Pump Genes and Virulence Determinants in *Enterococcus* spp. from Surface Water Systems. Environ. Sci. Pollut. Res..

[36] Maurya A.P., Rajkumari J., Bhattacharjee A., Pandey P. (2020). Development, Spread and Persistence of Antibiotic Resistance Genes (ARGs) in the Soil Microbiomes through Co-Selection. Rev. Environ. Health.

[37] Torres C., Alonso C.A., Ruiz-Ripa L., León-Sampedro R., Del Campo R., Coque T.M. (2018). Antimicrobial Resistance in *Enterococcus* spp. of Animal Origin. Microbiol. Spectr..

[38] Esfahani S., Ahmadrajabi R., Mollaei H., Saffari F. (2020). Co-Incidence of Type II Topoisomerase Mutations and Efflux Expression in High Fluoroquinolone Resistant *Enterococcus faecalis* Isolated from Urinary Tract Infections. Infect. Drug Resist..

[39] Ono S., Muratani T., Matsumoto T. (2005). Mechanisms of Resistance to Imipenem and Ampicillin in *Enterococcus faecalis*. Antimicrob. Agents Chemother..

[40] Kumari P., Tripathi B.M., Eo K.Y., Kimura J., Yamamoto N. (2025). Spatioseasonal Comparison of Fecal Resistome and Pathogenome of Raccoon Dogs in Korea. Ecohealth.

[41] Soares R.O., Fedi A.C., Reiter K.C., Caierão J., D’Azevedo P.A. (2014). Correlation between Biofilm Formation and *gelE*, *esp*, and *agg* Genes in *Enterococcus* spp. Clinical Isolates. Virulence.

[42] Madani W.A.M., Ramos Y., Cubillos-Ruiz J.R., Morales D.K. (2024). Enterococcal-Host Interactions in the Gastrointestinal Tract and Beyond. FEMS Microbes.

[43] Monteiro Marques J., Pita B., Pinto D., Barreto-Crespo M.T., Mato R., Semedo-Lemsaddek T. (2026). One Health Insights into *Enterococcus*: Antimicrobial Resistance and Virulence in Companion Animals and Their Tutors. Int. J. Mol. Sci..

[44] Erell E. (2017). Urban Greening and Microclimate Modification. Advances in 21st Century Human Settlements.

[45] Silva I., Alves M., Malheiro C., Silva A.R.R., Loureiro S., Henriques I., González-Alcaraz M.N. (2022). Short-Term Responses of Soil Microbial Communities to Changes in Air Temperature, Soil Moisture and UV Radiation. Genes.

[46] Hellberg R.S., Chu E. (2016). Effects of Climate Change on the Persistence and Dispersal of Foodborne Bacterial Pathogens in the Outdoor Environment: A Review. Crit. Rev. Microbiol..

[47] Thomson K., Berry R., Robinson T., Brown H., Bambra C., Todd A. (2020). An Examination of Trends in Antibiotic Prescribing in Primary Care and the Association with Area-Level Deprivation in England. BMC Public Health.

[48] Singer R., Sandfort M., Reichert F., Dörre A., Hoebel J., Klingeberg A., Haller S., Michalski N. (2025). Socioeconomic Position and Urban Environments as Drivers of Antimicrobial Resistance? An Ecological Study in Germany, 2010 to 2019. Eurosurveillance.

[49] Duan Y., Cole J., Mkrtchyan H.V., Xu Z. (2025). The Impact of Green Spaces, Urban Settings, Seasonal Changes, and Pollutants on Dissemination of Antimicrobial Genes in Air. Sci. Rep..

[50] CLSI (2023). Performance Standards for Antimicrobial Susceptibility Testing.

[51] EUCAST (2023). Breakpoint Tables for Interpretation of MICs and Zone Diameters, Version 13.0.

[52] Dutka-Malen S., Evers S., Courvalin P. (1995). Detection of Glycopeptide Resistance Genotypes and Identification to the Species Level of Clinically Relevant Enterococci by PCR. J. Clin. Microbiol..

[53] Arias C.A., Robredo B., Singh K.V., Torres C., Panesso D., Murray B.E. (2006). Rapid Identification of *Enterococcus hirae* and *Enterococcus durans* by PCR and Detection of a Homologue of the *E. hirae mur-2* Gene in *E. durans*. J. Clin. Microbiol..

[54] Sutcliffe J., Tait-Kamradt A., Wondrack L. (1996). *Streptococcus pneumoniae* and *Streptococcus pyogenes* Resistant to Macrolides but Sensitive to Clindamycin: A Common Resistance Pattern Mediated by an Efflux System. Antimicrob. Agents Chemother..

[55] Robredo B., Singh K.V., Baquero F., Murray B.E., Torres C. (2000). Vancomycin-Resistant Enterococci Isolated from Animals and Food. Int. J. Food Microbiol..

[56] Aarestrup F.M., Agerso Y., Gerner-Smidt P., Madsen M., Jensen L.B. (2000). Comparison of Antimicrobial Resistance Phenotypes and Resistance Genes in *Enterococcus faecalis* and *Enterococcus faecium* from Humans in the Community, Broilers, and Pigs in Denmark. Diagn. Microbiol. Infect. Dis..

[57] Aminov R.I., Garrigues-Jeanjean N., Mackie R.I. (2001). Molecular Ecology of Tetracycline Resistance: Development and Validation of Primers for Detection of Tetracycline Resistance Genes Encoding Ribosomal Protection Proteins. Appl. Environ. Microbiol..

[58] Mannu L., Paba A., Daga E., Comunian R., Zanetti S., Duprè I., Sechi L.A. (2003). Comparison of the Incidence of Virulence Determinants and Antibiotic Resistance between *Enterococcus faecium* Strains of Dairy, Animal and Clinical Origin. Int. J. Food Microbiol..

[59] Câmara Municipal do Porto: Portal de Dados. https://opendata.porto.digital/.

[60] Castro M.R., Alves H.T., Moreira C., Astell-Burt T., Cancela M., Guilherme F., Ribeiro A.I. (2026). Exposure to Green, Blue, and Biodiverse Spaces and Their Associations with Loneliness in Urban Adults: Findings from the EPIPorto Cohort. Health Place.

[61] Paciência I., Moreira A., Moreira C., Cavaleiro Rufo J., Sokhatska O., Rama T., Hoffimann E., Santos A.C., Barros H., Ribeiro A.I. (2021). Neighbourhood Green and Blue Spaces and Allergic Sensitization in Children: A Longitudinal Study Based on Repeated Measures from the Generation XXI Cohort. Sci. Total Environ..

[62] Ribeiro A.I., Launay L., Guillaume E., Launoy G., Barros H. (2018). The Portuguese Version of the European Deprivation Index: Development and Association with All-Cause Mortality. PLoS ONE.

[63] WHO (2016). Urban Green Spaces and Health.

